# Acute Appendicitis Presenting as Mechanical Small Bowel Obstruction in a Patient With Prior Abdominal Surgery: A Diagnostic Dilemma

**DOI:** 10.7759/cureus.105090

**Published:** 2026-03-12

**Authors:** Nehemia I Kassa, Filagot B Mikru, Yared Wasihun, Bewuketu Kefyalew, Bethel D Demissie

**Affiliations:** 1 Surgery, Myungsung Christian Medical Center, Addis Ababa, ETH; 2 General Surgery, Myungsung Christian Medical Center, Addis Ababa, ETH; 3 Radiology, Myungsung Christian Medical Center, Addis Ababa, ETH

**Keywords:** acute appendicitis, diagnostic dilemma, mechanical small bowel obstruction, perforated appendix, postoperative adhesions, previous abdominal surgery

## Abstract

Acute appendicitis is a common surgical emergency. However, its atypical presentations, such as mechanical small bowel obstruction (SBO), are exceedingly rare and diagnostically challenging. This is particularly true in patients with prior abdominal surgery, where postoperative adhesion is the most clinically significant cause. We report a 31-year-old man with a history of previous open gastro-jejunostomy who presented with progressive abdominal pain, distension, vomiting, and obstipation. Initial suspicion favored adhesive SBO; however, contrast-enhanced CT revealed a distended, inflamed appendix with marked peri-appendiceal inflammation causing distal ileal obstruction, distinct from the previous surgical site. Urgent exploratory laparotomy confirmed dense adhesions tethering the ileum to the inflamed appendix, which was successfully managed with adhesiolysis, release of the entrapped ileal segment, and appendectomy. The patient recovered uneventfully. This case underscores that prior abdominal surgery should not preclude consideration of appendicitis as a possible primary cause of mechanical SBO, particularly in patients with clinical features suggestive of an inflammatory process localized to the right lower quadrant. Additionally, it highlights the pivotal role of CT in differentiating inflammatory from adhesive obstruction and emphasizes the need for prompt surgical intervention to achieve favorable outcomes.

## Introduction

Acute appendicitis remains the most common surgical emergency worldwide, affecting approximately 7%-8% of the population during their lifetime [1]. The classical presentation of periumbilical pain migrating to the right lower quadrant is well established, yet appendicitis is notorious for its clinical variability [2]. Among its atypical manifestations, intestinal obstruction represents a particularly challenging presentation that can delay diagnosis and increase morbidity [3].

Appendicitis may produce either functional obstruction due to localized peritoneal inflammation or, far less commonly, true mechanical obstruction caused by inflammatory adhesions, abscesses, appendiceal phlegmons, or fibrous bands [4]. Mechanical small bowel obstruction (SBO) secondary to appendicitis is exceedingly rare, accounting for less than 1% of all SBO etiologies, and is almost exclusively reported in patients without prior abdominal surgery [5,6]. In patients with a history of abdominal interventions, postoperative adhesions are usually presumed to be the cause of obstruction, which can delay recognition of underlying appendiceal pathology [7]. This makes the occurrence of appendicitis-induced mechanical SBO in postoperative patients particularly uncommon and diagnostically challenging.

The diagnostic dilemma is compounded by overlapping clinical features, as both conditions present with abdominal pain, distension, vomiting, and obstipation. However, management pathways diverge significantly. Postoperative adhesive SBO may initially be managed conservatively, whereas appendicitis-induced mechanical obstruction typically requires urgent surgical intervention to address the underlying infectious process and prevent progression to perforation, peritonitis, and bowel ischemia [2,8]. We present a rare case of perforated appendicitis manifesting as mechanical SBO in a patient with a history of prior open gastro-jejunostomy, demonstrating that prior abdominal surgery should not prematurely close the diagnostic window for appendiceal pathology.

## Case presentation

A 31-year-old male patient with no known medical comorbidities presented to the emergency department with abdominal pain of two days' duration. The pain initially began in the periumbilical region and later localized to the lower abdomen. It was cramping in character and associated with anorexia, chills, and subjective low-grade fever. Twelve hours prior to presentation to the emergency department, he developed progressive abdominal distension, nausea, and two episodes of non-bilious vomiting. He also reported an absence of flatus and bowel movements for 24 hours prior to presentation. His surgical history was significant for an elective open gastro-jejunostomy for peptic gastric outlet stricture performed three years earlier, from which he recovered uneventfully.

On examination, he appeared acutely ill but was hemodynamically stable with a blood pressure of 120/70 mmHg, pulse of 99 bpm, temperature of 37.9°C, and respiratory rate of 16/min. The abdomen was mildly distended with hyperactive bowel sounds and hypertympanicity to percussion. Palpation revealed mild generalized tenderness with maximal intensity in the right lower quadrant and suprapubic region. This was accompanied by localized guarding, rigidity, and rebound tenderness in the right lower abdomen. The psoas sign was notably present. The previous surgical scar over the supraumbilical region appeared well-healed and non-tender.

Differential diagnoses at presentation included postoperative adhesive SBO, acute appendicitis, ileocecal inflammatory pathology, and, less likely, obstructing neoplasm or internal hernia. Given his prior surgery, initial consideration favored adhesive obstruction, but clinical features such as migratory abdominal pain, right lower quadrant tenderness, and systemic inflammatory response prompted evaluation for alternative etiologies.

**Figure 1:**
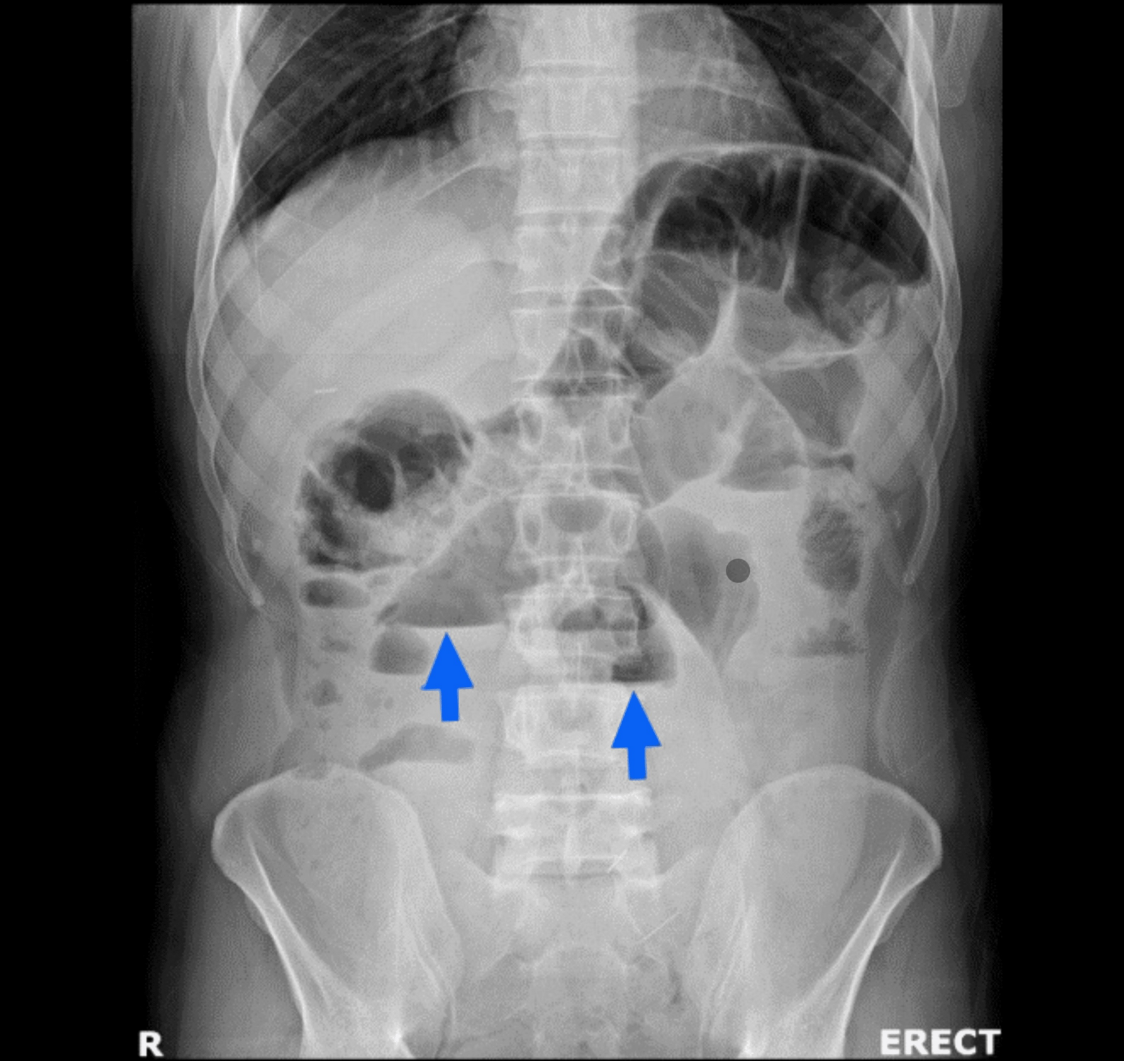
Erect plain abdominal radiograph demonstrating centrally located dilated small bowel loops with multiple air-fluid levels (blue arrows), suggestive of small bowel obstruction.

Laboratory tests showed leukocytosis (14,000/mm³ with 80% neutrophils) and elevated C-reactive protein (200 mg/L) (Table 1). Other laboratory tests were within normal limits. Plain abdominal radiography demonstrated dilated small bowel loops with multiple air-fluid levels, confirming SBO but providing no etiologic information (Figure 1). The patient was initially investigated with an abdominal ultrasound, which showed inflammatory changes and minimal free fluid in the right lower quadrant, but the appendix could not be visualized because of overlying bowel gas.

**Table 1:** Patient’s preoperative laboratory test results. Reference values were obtained from standard laboratory reference ranges [9]. WBC: white blood cell; BUN: blood urea nitrogen

Investigation	Patient’s result	Reference values
WBC count	14,000/µL	5,000-10,000/µL
Hemoglobin	14.7 g/dL	13-17 g/dL
Platelet count	250,000/µL	150-400,000/µL
C-reactive protein	200 mg/L	<10 mg/L
Kidney profile: BUN	10 mg/dL	7-20 mg/dL
Kidney profile: creatinine	0.8 mg/dL	0.7-1.3 mg/dL

**Figure 2:**
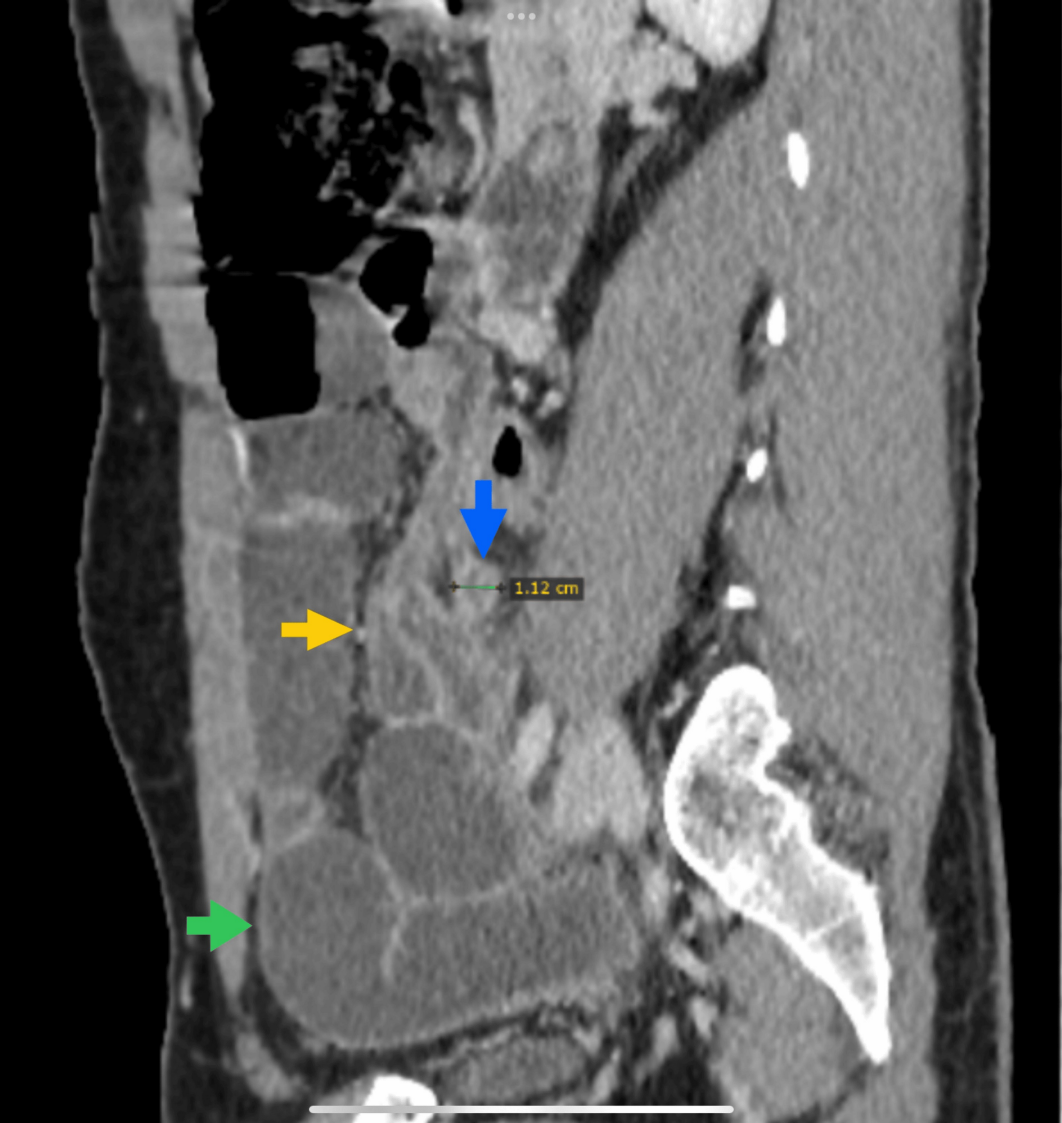
Sagittal contrast-enhanced CT image showing multiple dilated, fluid-filled small bowel loops (green arrow) with a transition point at the terminal ileum. The appendix is enlarged and hyperenhancing (1.12 cm, blue arrow) with surrounding peri-appendiceal inflammatory changes (yellow arrow).

Given the history of prior abdominal surgery, initial diagnostic consideration was given to postoperative adhesive SBO. However, the presence of fever, localized right lower quadrant tenderness, and elevated inflammatory markers prompted contrast-enhanced CT imaging. CT demonstrated a thick-walled, dilated appendix measuring 1.12 cm in diameter with marked peri-appendiceal fat stranding and minimal fluid collection (Figure 2). No appendicolith or focal defect in the appendiceal wall was identified. A transition point was noted at the terminal ileum, where an ileal loop was compressed by the surrounding peri-appendiceal inflammatory process. Proximal small bowel loops were markedly dilated, consistent with mechanical SBO. No adhesions or other abnormalities were detected at the site of the patient’s prior surgery, and no free intraperitoneal air was observed.

**Figure 3:**
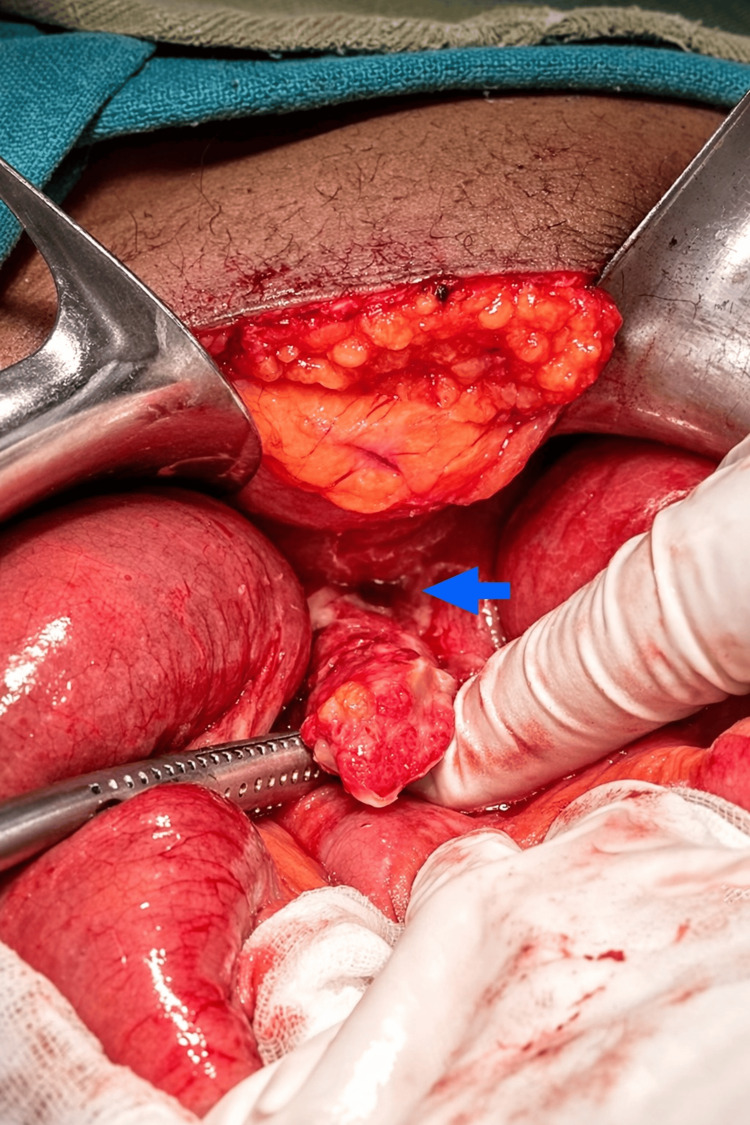
An inflamed appendix with a perforation at its base (blue arrow) identified after adhesiolysis, surrounded by marked inflammatory changes.

The patient was resuscitated with intravenous fluids, nasogastric decompression was performed, and broad-spectrum antibiotics (ceftriaxone plus metronidazole) were initiated. Given the history of prior abdominal surgery with the potential for intra-abdominal adhesions and the significant periappendiceal inflammatory process with suspected adhesion and perforation, an open approach was favored to allow safe adhesiolysis, thorough exploration, and definitive management. Urgent exploratory laparotomy was therefore performed through a midline incision. Intraoperative findings included approximately 50 mL of localized purulent fluid in the right iliac fossa. The proximal small bowel was markedly dilated, and a distal ileal loop was densely adherent to an inflamed appendiceal mass involving the cecum and adjacent omentum. The adhered bowel segment was viable with no evidence of ischemia. Notably, no adhesions were identified at the prior gastro-jejunostomy site, confirming that the obstruction was exclusively attributable to the appendiceal inflammatory process. Following careful adhesiolysis, the appendix was identified with significant inflammation and perforation at its base (Figure 3). Subsequently, the adhered ileal loop was freed, and appendectomy was completed without complications. The abdomen was closed after thorough lavage, and a suction drain was placed in the right lower quadrant.

Histopathological examination confirmed acute suppurative appendicitis with transmural inflammation, perforation, and micro-abscess formation. The postoperative course was uneventful. Oral feeding was initiated on postoperative day one, and bowel function returned on day two, after which the nasogastric tube was removed. The suction drain was removed on postoperative day four after minimal output, and the patient was discharged on postoperative day five. Antibiotics were continued for a total of seven days after the surgery. At the three-month follow-up, he remained asymptomatic with complete resolution of symptoms.

## Discussion

This case highlights an important diagnostic challenge. Prior abdominal surgery can bias clinicians toward attributing SBO to postoperative adhesions, potentially delaying recognition of alternative causes that require different management strategies. Although adhesions account for approximately 60%-75% of SBO cases in patients with previous surgery [10], this statistical predilection should not preclude consideration of other etiologies when clinical features suggest an acute inflammatory process. In our patient, the history of open gastro-jejunostomy initially favored adhesive obstruction. However, several clinical features suggested an alternative etiology, including progression from migratory abdominal pain to obstructive symptoms, persistent right lower quadrant tenderness with peritoneal signs, systemic inflammatory response with fever and elevated inflammatory markers, and the absence of prior similar obstructive episodes. Clinically, adhesive SBO typically presents abruptly in patients with prior surgery, often without preceding visceral pain, whereas appendicitis-induced obstruction generally follows initial migratory pain, localizes to the right lower quadrant, and is frequently accompanied by systemic inflammatory signs [4,11]. Imaging can further support differentiation because in adhesive SBO, the transition point is usually at a prior surgical site or fibrous band with no surrounding inflammation, whereas appendicitis-induced SBO shows an enlarged appendix, peri-appendiceal fat stranding, and sometimes abscess formation near the transition point [4]. These findings underscore the importance of integrating clinical course, physical examination, and imaging rather than relying solely on surgical history in determining the cause of SBO.

The pathophysiology of mechanical SBO secondary to appendicitis most commonly involves inflammatory adhesion formation, where fibrinous exudate from peritoneal inflammation organizes into fibrous bands tethering ileal loops to the appendix or adjacent abscess cavity. Less frequently, external compression may occur from an appendiceal phlegmon or mass, and rare mechanisms such as appendico-ileal knotting have been described [5,11]. In this case, perforation triggered an intense inflammatory response with fibrin deposition, leading to dense adhesions between the terminal ileum and the appendiceal mass. This process is consistent with the pathophysiology of adhesion formation, where initial fibrinous exudate progresses to fibroblast proliferation and collagen deposition, creating permanent fibrous bands capable of mechanical obstruction [12]. The absence of adhesions at the prior surgical site confirmed that the obstruction was driven by appendiceal inflammation rather than postoperative changes.

When clinical assessment cannot clearly distinguish between adhesive SBO and inflammatory causes, contrast-enhanced CT is indispensable. CT allows accurate localization of the transition point and identification of underlying pathology, including appendiceal enlargement exceeding 6 mm, wall thickening, hyperenhancement, peri-appendiceal fat stranding, and abscess formation [13]. The sensitivity of CT for diagnosing appendicitis approaches 95% when performed with intravenous contrast [14], and in this patient, CT was pivotal in establishing the diagnosis and facilitating timely operative management. The demonstration of inflammatory changes directly adjacent to the transition point, with no abnormalities at the prior surgical site, provided critical diagnostic clarity.

Mechanical SBO caused by acute appendicitis is exceedingly rare, with only a handful of cases reported in the literature [5,6,15]. Notably, the vast majority of these cases occurred in patients without prior abdominal surgery. For example, Harrison et al. and Barazi et al. described appendicitis-induced SBO in surgically naïve patients, with obstruction typically resulting from inflammatory adhesions or the appendix directly impinging on the ileum [6,15]. Similarly, Assenza et al. reported a case in which an inflamed appendix wrapped around the terminal ileum, again in a patient with no prior abdominal operations [5]. The presence of previous surgery in our case, therefore, represents a key confounding factor and illustrates the risk of diagnostic anchoring. This case contributes to the limited literature by demonstrating that appendicitis should remain in the differential diagnosis of SBO even in patients with a surgical history, particularly when symptoms begin with migratory pain or when inflammatory signs localize to the right lower quadrant.

Management differs fundamentally from uncomplicated adhesive SBO, which may be treated conservatively with nasogastric decompression and observation in selected stable patients. In contrast, obstruction secondary to appendicitis requires prompt surgical intervention because of the underlying infectious process and the risk of progression to generalized peritonitis or bowel compromise [8]. Definitive treatment includes appendectomy, adhesiolysis, and drainage of any abscess, with careful assessment of bowel viability. Early recognition and intervention are essential and are associated with favorable outcomes, as demonstrated in this case, where the patient recovered uneventfully and remained asymptomatic at follow-up.

Several clinical lessons emerge from this case. First, a history of prior abdominal surgery should not exclude acute appendicitis as a cause of mechanical SBO, particularly when obstructive symptoms follow typical appendiceal pain or inflammatory signs localize to the right lower quadrant. Second, temporal symptom progression is important, as appendicitis-induced obstruction typically follows an initial period of pain, whereas adhesive SBO often presents abruptly. Third, a low threshold for CT imaging is recommended in patients with suspected atypical or alternative causes of obstruction, as it helps clarify the etiology and guide surgical planning.

## Conclusions

Mechanical SBO due to acute appendicitis is rare, particularly in patients with prior abdominal surgery, where adhesions are often presumed to be the cause. This case demonstrates that postoperative adhesive disease should not be assumed as the default etiology of SBO, particularly when clinical features suggest an inflammatory process localized to the right lower quadrant. Contrast-enhanced CT plays a key role in establishing the diagnosis, and prompt surgical management leads to favorable outcomes. Clinicians should maintain a high index of suspicion for appendiceal pathology in any patient with SBO accompanied by fever, localized tenderness, and elevated inflammatory markers, regardless of surgical history.
